# Ossiculoplasty in Cholesteatoma: An Evaluation of Necessity Versus Discretion in a Tertiary Center Retrospective Analysis

**DOI:** 10.7759/cureus.101954

**Published:** 2026-01-20

**Authors:** Michael Rezk, Mostafa Sordy, Khaled Ghasnawy, Mina F Saleeb, Ossama M Mady

**Affiliations:** 1 Department of Otolaryngology, Ain Shams University, Cairo, EGY; 2 Department of Otorhinolaryngology, Ain Shams University, Cairo, EGY

**Keywords:** cholesteatoma, com, hearing outcome, mastoid obliteration, ossiculoplasty

## Abstract

Background: While the main aim of cholesteatoma surgery is to eradicate the disease and minimize the risk of recurrence, achieving the best possible hearing after surgery remains a key secondary objective, one that matters greatly to patients. Predicting an individual’s postoperative hearing outcome is difficult because it depends not only on factors such as the condition of the ossicular chain, the type of ossicular reconstruction, and the extent of the cholesteatoma, but also potentially on other influences, including middle-ear aeration, which has been suggested to affect hearing results.

Aim: This study aimed to assess hearing outcomes, measured as air-bone gap (ABG) gain, in canal wall down mastoidectomy (CWDM) patients who underwent ossiculoplasty with TORP or PORP, either concurrently or at six months postoperatively, with or without mastoid obliteration.

Methods: This retrospective cohort study reviewed all adult patients who underwent CWDM at a tertiary referral hospital between 2017 and 2024 and completed audiological follow-up at our institution. Hearing outcomes were evaluated using standardized audiometry at three time points: preoperative baseline, an early postoperative assessment, and a longer term postoperative follow-up. Changes in hearing measures over time were quantified, and we examined potential predictors of postoperative hearing performance, including patient- and surgery-related factors that might influence auditory recovery.

Results: Among 45 patients (62.2% men; mean age: 33.98 ± 10.8 years), baseline hearing averaged 47.5 ± 7.7 dB air conduction (AC), 19.9 ± 7.1 dB bone conduction (BC), and 27.5 ± 10.2 dB ABG. Age and pretreatment measures were nearly symmetric, whereas posttreatment AC/BC showed positive skewness (BC strongest). Groups I-IV did not differ significantly on any audiologic variable (all p values >0.05). Repeated analysis of covariance showed significant time effects for AC (p = 0.003; η_p_² = 0.195) and ABG (p < 0.001; η_p_² = 0.26), but not for BC; Wilcoxon tests still indicated a change for BC (p = 0.005). Assumptions were largely satisfied overall (Levene/Box).

Conclusions: CWDM with ossiculoplasty produced significant improvement in AC and ABG over time, with no differences by reconstruction type, timing, or mastoid obliteration. BC changes were limited, reflecting heterogeneous outcomes and warranting larger studies.

## Introduction

The primary goal of a mastoidectomy for chronic otitis media (COM) with cholesteatoma is the removal of lesions. Traditionally, the best treatment for cholesteatoma has been thought to be canal wall down mastoidectomy (CWDM) [1]. For these patients, satisfactory functional outcomes are necessary. Ossiculoplasty, or the repair of an intact ossicular chain, is frequently done concurrently with or after CWDM to improve hearing [2].

When the ossicular chain is disrupted, ossiculoplasty is typically deferred during CWDM if a staged procedure is planned or if there is a high likelihood of recurrence due to poor Eustachian tube function. In such cases, CWDM is performed along with cavity grafting instead [1]. Sound transmission has been preserved using a range of techniques and materials, such as incus transposition [3].

Since the first ossiculoplasty was introduced in the 1950s, numerous techniques have been developed to restore hearing in patients with COM, with or without cholesteatoma. The two-stage approach involves removing inflammatory tissue during the initial procedure, followed by ossiculoplasty after the middle ear has stabilized. This method has proven effective in enhancing postoperative hearing outcomes [4]. In this study, we aimed to analyze hearing outcomes, expressed as air-bone gap (ABG) gain, in CWDM patients who underwent ossiculoplasty with a total ossicular replacement prosthesis (TORP) or partial ossicular replacement prosthesis (PORP), either at the time of surgery or six months later, with or without mastoid obliteration.

## Materials and methods

We reviewed the records of CWDM at a tertiary center between 2017 and 2024. Eligible cases had chronic otomastoiditis with middle-ear cholesteatoma confirmed on CT and were managed with CWDM; CT showed no inner-ear anomalies. Patients with preoperative BC thresholds >65 dB hearing level (HL) were excluded. All procedures were performed by two surgeons. Ossicular reconstruction was carried out with a PORP or TORP, depending on the presence of the stapes suprastructure, and mastoid obliteration was performed using tragal cartilage or bone chips.

Patients were divided into four groups: 1) ossiculoplasty performed at the time of CWDM without mastoid obliteration, 2) ossiculoplasty performed at the time of CWDM with mastoid obliteration, 3) ossiculoplasty performed six months after CWDM without mastoid obliteration, and 4) ossiculoplasty performed six months after CWDM with mastoid obliteration.

Surgical technique

**Figure 1:**
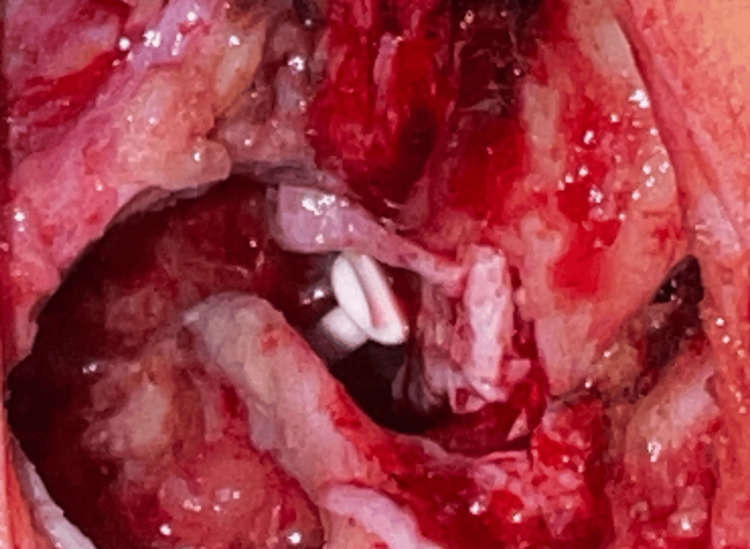
Reconstruction by cartilage and bone chips

A postauricular method was used, harvesting superficial and deep temporalis fascia grafts and elevating a wide tympanomeatal flap to access the middle ear, with canaloplasty as required. After complete mastoidectomy, bone pâté was collected from the healthy cortex for later use. Cholesteatoma and granulation tissue were thoroughly removed. The attic and upper third of the posterior canal wall were drilled while preserving the lower two-thirds to maintain support and fully expose the epitympanum. The malleus and incus remnants were excised, and residual disease was carefully cleared from the epitympanum, stapes, oval window, and facial canal; the sinus tympani was inspected endoscopically to minimize recurrence. Conchal cartilage was then harvested, deperichondrialized, split, and used to reconstruct the attic and the superior posterior canal wall (Figure 1).

The mastoid cavity and epitympanum were meticulously obliterated using tragal cartilage or bone chips harvested during mastoidectomy. The collected bone pâté also provided structural support for the reconstructed posterior canal wall. All patients underwent primary ossicular reconstruction with either a partial or TORP, selected according to the presence or absence of the stapes suprastructure, and this was performed even when a planned second-look procedure was anticipated. The prosthesis helped preserve middle-ear space and buttress the reconstructed cartilage wall. The superficial temporalis fascia layer was used as a soft-tissue cover over the conchal cartilage, whereas the deep temporalis fascia was used to recreate the tympanic membrane. Antibiotic- and steroid-soaked Gelfoam (Pfizer, Kalamazoo, MI) was placed both medial and lateral to the newly formed membrane.

Meatoplasty was not undertaken. At the end of the procedure, the external auditory canal was gently packed with gauze impregnated with antibiotic ointment. The postauricular incision was then closed in two layers, and a routine mastoid pressure dressing was applied. Postoperatively, patients received an oral quinolone antibiotic course for one week as prophylaxis against infection. The Gelfoam packing was removed on postoperative day 7. Follow-up visits were scheduled weekly, with healing and cavity status assessed using otoendoscopy. Beginning two weeks after surgery, patients were instructed to perform regular Valsalva maneuvers to evaluate tympanic membrane mobility clinically, promote middle-ear aeration, and reduce the risk of tympanic membrane retraction.

Analysis of surgical results and audiological tests

Postoperative evaluation of the tympanic membrane was carried out using both endoscopic and microscopic techniques. Any complications, such as dizziness, facial nerve palsy, tympanic membrane reperforation, wound infection, including myringitis, or prosthesis extrusion, were documented. A hearing evaluation was carried out utilizing pure-tone audiometry, with the pure-tone average (PTA) determined by the four-frequency method (0.5, 1, 2, and 4 kHz). The thresholds for both air conduction (AC) and bone conduction (BC) were measured prior to and following surgery, and the ABG was calculated. For patients with multiple postoperative audiograms, the most recent result was used. The primary outcome measure was improvement in ABG, defined as the change in PTAs between pre- and postoperative tests. Ossiculoplasty was deemed successful when the postoperative ABG was ≤20 dB.

## Results

The study included 45 patients, with female participants comprising 37.8% and male participants 62.2%. The average age of the participants was 33.98 ± 10.8 years. The mean baseline HLs were 47.5 ± 7.7 dB as measured by AC and 19.9 ± 7.1 dB by bone conduction (BC). The mean baseline ABG was 27.5 ± 10.2 dB. The age distribution shows slight positive skewness, but the skewness ratio suggests it is relatively symmetric. Pretreatment AC and ABG have minor negative skewness, with their ratios indicating moderate skewness within acceptable limits. Posttreatment AC and BC exhibit moderate-to-strong positive skewness, particularly in BC, where the high skewness ratio indicates a significant departure from normality, potentially reflecting varied treatment outcomes. Pretreatment BC also shows moderate positive skewness, requiring careful interpretation. Posttreatment ABG has mild positive skewness, with a ratio suggesting it is not extreme and generally acceptable for most analyses (Table 1).

**Table 1:** Age and hearing levels of studied cases Data are presented as mean ± SD. Between-group comparisons were performed using one-way ANOVA (F test). Statistical significance was set at p < 0.05 (two-tailed) SD: standard deviation; SE: standard error; AC: air conduction; BC: bone conduction; ABG: air-bone gap; ANOVA: analysis of variance

Parameter	Min.	Max.	Mean	SD	Skewness statistic	SE	Ratio	Skewness
Age	17	53	33.98	10.769	0.202	0.354	0.57	Slight
AC (pre)	30	60	47.549	7.725	-0.452	0.354	-1.28	Slight
AC (post)	15	70	32.51	12.06761	1.294	0.354	3.66	Moderate
BC (pre)	10	44	19.867	7.0866	1.42	0.354	4.01	Moderate
BC (post)	10	70	18.4556	12.3088	2.645	0.354	7.47	Strong
ABG (pre)	5	43	27.46	10.2011	-0.51	0.354	-1.44	Slight
ABG (post)	0	35	13.8322	6.89509	0.551	0.354	1.56	Mild

Comparisons among the four groups (I-IV) across various audiological measures reveal no significant differences. Age, AC (pre and post), BC (pre and post), and ABG (pre and post) all have p values well above 0.05, indicating that the mean differences across groups are not statistically significant. The F-statistics further suggest minimal variance between the groups, reinforcing their similarity in these parameters (Table 2).

**Table 2:** Comparison between studied groups as regards the hearing levels Data are presented as mean ± SD. Between-group comparisons were performed using one-way ANOVA (F test). Statistical significance was set at p < 0.05 (two-tailed) SD: standard deviation; AC: air conduction; BC: bone conduction; ABG: air-bone gap; ANOVA: analysis of variance

Men (%)	Group I (n = 12)		Group II (n = 10)		Group III (n = 13)		Group IV (n = 10)		F statistic	p value
	Mean	SD	Mean	SD	Mean	SD	Mean	SD		
Age	33.00	10.03	34.00	11.59	35.15	10.46	33.60	12.67	0.083	0.969
AC (pre)	46.93	7.60	45.25	8.87	49.46	9.15	48.10	4.33	0.586	0.628
AC (post)	29.83	12.38	31.70	13.81	34.00	9.46	34.60	14.01	0.362	0.781
BC (pre)	18.42	5.98	21.35	6.26	21.08	8.25	18.55	7.91	0.537	0.659
BC (post)	16.71	7.79	21.15	14.25	17.19	9.66	19.50	17.98	0.760^K^	0.859
ABG (pre)	28.52	7.92	22.90	10.29	28.39	11.79	29.55	10.49	0.877	0.461
ABG (post)	13.12	5.82	10.55	6.08	16.04	5.62	15.10	9.52	1.39	0.259

We assessed HLs using a conventional audiogram with three different measures: AC, BC, and ABG. For AC, the time effect is significant with a p value of 0.003 and an η_p_² of 0.195, indicating a moderate effect on HLs over time. However, the interactions between time-age and time-group are not significant, suggesting that these factors do not significantly impact the effect of time on HLs. In BC, none of the factors or interactions are significant, as evidenced by p values above 0.05, indicating no substantial effect of time, age, or group on BC. For ABG, the time effect is highly significant (p < 0.001) with a large effect size (η_p_² = 0.26), implying a strong impact of time on the ABG. The other factors and interactions for ABG are not significant, suggesting that age and group do not significantly influence the changes in ABG over time. Levene's test and Box's test results indicate that the assumptions of homogeneity of variances and covariance are generally met (Table 3).

**Table 3:** Two-way repeated ANCOVA test for the hearing levels Two-way repeated-measures ANCOVA was used (within-subject factor: time (pre vs. post); between-subject factor: group; covariate: age). Effect size is reported as partial η². Assumptions were assessed using Levene’s test (homogeneity of variances) and Box’s M test (homogeneity of covariance matrices). Statistical significance was set at p < 0.05 (two-tailed) For BC (pre vs. post), the Wilcoxon signed-rank test was additionally applied due to nonnormal distribution (p = 0.005) ^*^Statistical significance AC: air conduction; BC: bone conduction; ABG: air-bone gap; ANCOVA: analysis of covariance

Parameter	F	p value	η_p_^2^	Levene's test (pre)	Levene's test (post)	Box's test
AC						
Time	9.7	0.003^*^	0.195	0.104	0.574	0.391
Age	0.102	0.751	0.003			
Group	0.642	0.592	0.046			
Time-age	1.048	0.312	0.026			
Time-group	0.164	0.920	0.012			
BC						
Time	0.99	0.326	0.024	0.941	0.396	0.004^*^
Age	0.213	0.647	0.005			
Group	0.26	0.854	0.019			
Time-age	0.512	0.478	0.013			
Time-group	0.87	0.465	0.061			
ABG						
Time	14.058	<0.001^*^	0.26	0.385	0.830	0.213
Age	0.573	0.454	0.014			
Group	1.31	0.284	0.089			
Time-age	0.685	0.413	0.017			
Time-group	0.285	0.836	0.021			

## Discussion

CWDM is a safe, dependable operation in experienced hands and is widely used to achieve durable control of squamosal COM with cholesteatoma, especially when attic or mastoid disease is extensive and complete clearance is crucial to reduce recurrence. Historically, the main drawback was the limited expectation of postoperative hearing improvement because cholesteatoma and surgical eradication often disrupt the ossicular chain and alter middle-ear mechanics. In recent decades, this outlook has improved substantially. Advances in tympanoplasty and ossiculoplasty, along with improved reconstructive principles, have made functional hearing restoration increasingly achievable alongside disease eradication; consequently, CWDM is now frequently integrated into reconstructive plans to optimize conductive hearing when possible. A commonly used reconstructive method is incus interposition, in which a reshaped incus remnant is placed to bridge the malleus handle and stapes suprastructure, particularly when the incus is eroded. Ossicular damage patterns are predictable: long-process incus necrosis is most frequent, followed by complete incus loss with preserved stapes suprastructure; the most severe cases leave only the stapes footplate, often requiring total ossicular replacement [5].

In this study, we demonstrated that the comparisons among the four groups (I-IV) across various audiological measures show no significant differences. Age, AC (pre and post), BC (pre and post), and ABG (pre and post) all have p values well above 0.05, indicating that the mean differences across groups are not statistically significant. The F-statistics further suggest minimal variance between the groups, reinforcing their similarity in these parameters.

Mathur et al. [6] assessed mastoid cavity obliteration using postauricular soft tissue in patients undergoing CWDM. They reported that, compared with the conventional nonobliterative technique, sealing the cavity with postauricular soft tissue was associated with improved clinical outcomes, most notably faster postoperative healing and a lower incidence of persistent otorrhea/discharge, suggesting that obliteration may reduce common cavity-related problems and enhance overall recovery.

Meta-analysis by Salem et al. [7] of 12 studies (2,379 patients) reported comparable recurrence rates (OR: 1.231, 95% CI: 0.550-2.757, p = 0.613). However, the canal wall down with mastoid obliteration (CWD-MO) showed lower recurrence rates compared to canal wall up (CWU) mastoidectomy (OR: 0.330, p < 0.001).

A comparative study by Wilkie et al. [8] reported otorrhea rates of 7.3% for CWD-MO vs. 10.2% for CWD, although this difference was not statistically significant. Van der Toom et al. [9] conducted a comparative analysis of hearing outcomes following CWDM with mastoid obliteration vs. nonobliteration CWU and CWD techniques in a large cohort at their tertiary referral center. Given the association between middle-ear aeration and hearing results, they hypothesized that obliteration might yield superior postoperative hearing compared to nonobliteration approaches. However, their findings revealed no significant differences among the techniques, noting that such comparisons are complex due to the multiple factors influencing postoperative hearing. Likewise, Erfurt et al. [10] reported no significant differences between the two groups regarding postoperative BC thresholds (mean difference: 2.7 dB, p = 0.221) or ABG closure at six weeks (2.3 dB in the nonobliteration group vs. 1.5 dB in the obliteration group, p = 0.903).

Mastoid cavity obliteration was initially described by Mosher [11] and was later refined and widely promoted during the 1960s by Palva [12]. After Mercke [13] reported favorable results using obliteration in CWD cholesteatoma surgery, the approach gained broader acceptance and was progressively adapted by many otologists. As a result, the literature now describes numerous obliteration methods employing a range of materials, reflecting ongoing efforts to optimize long-term cavity outcomes.

Across these variations, the shared objective is to preserve the low recidivism rates traditionally associated with CWD mastoidectomy while reducing the well-known postoperative “cavity issues,” such as chronic discharge, debris accumulation, and prolonged healing [14]. However, no single technique is ideal for every case. Each option carries specific trade-offs related to material resorption, volume loss/atrophy, ease of shaping and contouring, donor-site morbidity, and susceptibility to infection [15].

In this study, we found that for AC, the time effect is significant with a p value of 0.003 and an η_p_² of 0.195, indicating a moderate effect on HLs over time. Additionally, for ABG, the time effect is highly significant (p < 0.001) with a large effect size (η_p_² = 0.26), indicating a strong impact of time on ABG.

Walker et al. [16] reported a mean preoperative ABG of 27.8 dB in their overall cohort (n = 285). Most patients (253; 89%) proceeded to a planned second-look procedure with ossiculoplasty. Among these, 148 ears had postoperative audiometry available at one year or more after the second-look surgery. In this subgroup, the mean ABG was 23.4 ± 11.7 dB, representing an average improvement of approximately 4.2 dB relative to the preoperative ABG (27.6 ± 12.9 dB), indicating a modest but measurable gain in conductive hearing following staged reconstruction.

Kim et al. [17] assessed the outcomes of modified CWDM combined with mastoid obliteration using autologous materials. Among 76 patients who underwent a single-stage procedure and were evaluated with pure-tone audiometry at 12 months postoperatively, 14.5% achieved an ABG of 0-10 dB HL, 36.8% had 10-20 dB HL, 30.3% showed 20-30 dB HL, and 18.4% exceeded 30 dB HL. The mean ABG improved significantly from 26.7 ± 10.9 dB HL preoperatively to 20.8 ± 10.8 dB HL postoperatively (p = 0.001), indicating a substantial improvement in hearing outcomes following surgery.

Liu et al. [18] reported an average PTA improvement of 4.5 dB at one year and 2 dB at four years postoperatively. The mean ABG improvement was 4.4 dB at one year and 2.2 dB at four years, with the one-year change statistically significant (p < 0.05). At the one-year follow-up, 70.4% of patients achieved excellent (0-10 dB) or good (11-20 dB) gap closure, while 63.7% maintained these outcomes at four years. Both improvements were statistically significant compared with preoperative values (p < 0.05), although some patients continued to experience conductive hearing loss.

The outlook for mastoid cavity obliteration using postauricular soft tissue during CWDM is moving toward more refined techniques that aim to improve hearing-related quality of life, reduce cavity problems, and streamline surgery. One likely direction is the broader adoption of endoscopic and other minimally invasive approaches, either as stand-alone methods or as adjuncts to microscopy. Improved visualization, particularly of hidden recesses, can enhance precision during disease clearance and reconstruction, potentially lowering residual disease rates and reducing postoperative complications such as chronic discharge or debris accumulation. Smaller, more targeted dissection may also translate into less tissue trauma, faster healing, and shorter recovery. Another major area of progress is materials science. Future obliteration strategies may increasingly incorporate next-generation biomaterials designed to behave more like native tissues. Examples include bioactive scaffolds that support cell ingrowth, encourage vascularization, and promote predictable integration with surrounding structures. If these materials can provide durable volume stability while enhancing healing, they could improve long-term cavity outcomes and reduce the need for revision procedures. Overall, the trend is toward techniques and materials that make obliteration more reliable, less invasive, and more consistent across diverse patient anatomies [19].

The limitation of the study includes its retrospective design, which inherently introduces selection bias and relies on potentially incomplete medical records. The relatively small sample size of 45 patients, when stratified into four distinct subgroups, significantly limits the statistical power to detect subtle differences between surgical techniques. Consequently, the findings primarily reflect the experience of a single tertiary center and may not fully generalize to broader clinical settings. Furthermore, the lack of randomization prevents a definitive comparison between single-stage and staged procedures, necessitating cautious interpretation of the nonsignificant differences observed between the study groups. Additionally, the follow-up duration varied among patients, potentially obscuring long-term complications. Future research should prioritize large-scale, multicenter prospective randomized controlled trials to overcome the statistical limitations inherent in small retrospective cohorts.

## Conclusions

The findings of this retrospective cohort study confirm that CWDM combined with ossicular reconstruction is a highly effective procedure for restoring hearing function in patients with COM and cholesteatoma. The statistical analysis demonstrated a highly significant improvement in AC thresholds and ABG closure over time, affirming the functional benefits of the surgery.
